# Fracture resistance and pattern of the upper premolars with obturated canals and restored endodontic occlusal access cavities

**DOI:** 10.1016/S1674-8301(10)60063-2

**Published:** 2010-11

**Authors:** Younong Wu, Peter Cathro, Victor Marino

**Affiliations:** a Dental Research Institute, Nanjing Medical University, Nanjing, Jiangsu 210029, China; b School of Dentistry, Faculty of Health Sciences, University of Adelaide, SA 5005, Australia

**Keywords:** root canal preparation, premolar, fracture

## Abstract

We studied whether obturing canals and restoring endodontic occlusal access cavities on upper premolars could provide acceptable resistance and pattern to fracture. Eighteen upper premolars were divided equally into 3 groups. Group 1 consisted of intact controls; group 2 had access cavities and root canal preparations; group 3 as in group 2 but obturated with gutta-percha and AH26, and the access cavity restored with glass ionomer and composite. Specimens were submitted to compressive strength testing using the Hounsfield Universal H50KM testing machine with a load cell of 2000 Newtons and a crosshead speed set at 1.0 mm/min until fracture. The results from the compressive strength tests showed that intact controls (1105.83±90.93 MPa) and restored premolars (936.67±44.67 MPa) were significantly different from premolars with unrestored access cavities 568.33±105.49 MPa. There was no significant difference between intact controls and restored premolars. The predominant fracture pattern for intact teeth was an oblique fracture. For premolars that had endodontic access cavities, restored or unrestored, the most common fracture pattern was a vertical fracture. The restoration of occlusal access cavities with glass ionomer and composite provided fracture resistance close to that of intact controls, but when restored teeth fractured, they were predominantly non-restorable.

## INTRODUCTION

Teeth that have suffered pulp necrosis and apical periodontitis can be managed by conventional root canal therapy. Whilst the broad principles of endodontics have remained similar since 1826[1], there have been advances in instruments, materials and techniques to potentially improve the quality of endodontic treatment. However, the longevity of the tooth is often dictated by the coronal restoration and its ability to prevent leakage and resist fracture. If a tooth fractures favourably, then it may be possible to provide a complex restoration, but an unfavourable crown-root fracture may result in the need for extraction. In order to prevent the endodontically treated tooth from fracture, full cuspal protection with either an overlay restoration or crown is often recommended as soon as root canal therapy has been completed, especially for maxillary and mandibular premolars and molars[2].

The inherent elastic properties of intact enamel and dentine are altered when even just an occlusal cavity is prepared without endodontic access, creating a reduction in fracture resistance[3]. In order to gain entry to the root canal system, the endodontic access cavity cuts completely through the enamel and dentine in an apical direction, which significantly reduces the rigidity of the tooth[4]. With the removal of both marginal ridges in a mesial-occlusal-distal (MOD) cavity preparation and in conjunction with an endodontic access cavity, a dramatic increase in cuspal deflection is observed[5]. Tooth fracture resistance seems to be only partially recovered when MOD preparations are associated with an endodontic access and restored with composite resin[6],[7]. Howe and McKendry[8] using molar teeth found that an occlusal endodontic access significantly weakens the tooth compared to the intact controls. Interestingly, the authors found that occlusal endodontic access opening of the mandibular molar was not significantly weaker than a conservative MOD cavity preparation without endodontic access. The authors, therefore, questioned the concept that all endodontically treated posterior teeth should have cuspal protected restorations.

Few studies have been carried out on the premolars when the marginal ridges remain intact, with only an occlusal access cavity prepared. Steele and Johnson[9] using compressive fracture tests found that an endodontic cavity preparation in an otherwise intact tooth had a similar strength to that of an intact tooth. Reeh *et al.*[10] established that the loss of the marginal ridge integrity resulted in the greatest loss of stiffness. There seems to be little evidence to guide the clinician if only the access cavity needs restoring. This may occur in situations including occlusal caries, management of cases with dens-evaginatus and certain traumatic injuries.

Teeth are potentially at risk during root canal therapy (RCT) as the access cavity is only temporarily restored. Identification of these factors may help modify treatment practices and thereby reduce the chance of unrestorable fractures. The clinical dilemma remains whether or not they require cuspal protection at the completion of endodontic treatment.

The aims of this study were to evaluate the fracture resistance and pattern of the upper premolar teeth that have an occlusal access cavity restored with glass ionomer and composite, and to investigate fracture patterns.

## MATERIALS AND METHODS

### Sample collection

The collection of teeth for experimental purposes came with the approval from the Human Ethics Committee of the University of Adelaide.

Intact upper premolar teeth with one or two roots, completely formed apices and without visible signs of cracks or caries were chosen from a large pool of teeth collected from the Adelaide Dental Hospital. These were stored in 10% phosphate buffered formalin.

Radiographs were taken in the mesio-distal and bucco-palatal dimensions. Eighteen teeth identified with two canals were selected and then transferred to individually numbered containers filled with deionised water.

### Experimental groupings

The teeth were sorted into six lots of three teeth that had similar size, shape and root formation. This was done to help establish meaningful comparison between the testing protocols. Within each lot, the teeth were then randomly assigned to be either an intact control (group 1), or to have an access cavity and RCT preparation (group 2), or to have an access cavity, RCT preparation followed by obturation with gutta-percha and AH26 and the access cavity restored with glass ionomer and composite (group 3).

### Sample preparation

Group 1: The teeth in this group were untreated and served as intact controls.

Group 2: An oval access cavity was prepared in the centre of the occlusal surface between the cusps using a high-speed handpiece with carbide bur (#3131 FG, KG Sorensen Ind. Com, Brazil) until the two canal orifices were identified. A #10K hand file was inserted until the tip of the file just was visible at the apical foramen, and this length was measured with 0.5 mm deducted from this measurement to arrive at the working length. The teeth were prepared using rotary NiTi files (K3-SybronEndo) in a crown-down sequence until at least a 06 #25 file reached the working length. Deionised water was used to irrigate the canals between each file change.

Group 3: The teeth were prepared as in group 2 and then the canals were obturated with tapered gutta-percha cones ( Dentsply Maillefer), endodontic cement ( AH26 Dentsply) and accessory cones using the lateral condensation technique. After obturation the gutta-percha was reduced to the canal orifice using a heated plugger. The access cavities were then wiped with alcohol to remove excess sealer. The dentine was conditioned and then a 2 mm base of glass ionomer (Fuji IX) was applied. When the glass ionomer had set, each tooth was etched with 37% phosphoric acid solution (3M ESPE) for 30 sec around the cavity, then rinsed with water for 20 sec and dried with air jets. Primer and bonding adhesive (Scotchbond Multi-Purpose System, 3M ESPE) were applied according to the manufacturer's instructions and light-cured for 20 sec. The composite resin (Filtek Supreme XT, 3M ESPE) was placed into the cavity incrementally with each increment light-cured for 40 sec using a visible-light curing unit (XL2500, 3M; light intensity, 580 mW/cm^2^).

Two coats of Poyether Adhesive (3M ESPE) were applied to the root surfaces of all teeth using microbrushes to simulate the periodontal ligament. The adhesive was air dried and the teeth were stabilised vertically with the crown upwards by soft wax in plastic boxes (2.5 cm×2.5 cm×2.0 cm). Cold cure resin (Vertex) was mixed and then poured slowly into the plastic boxes until the surface of resin was 1 mm lower than the cementoenamel junction. After setting, the teeth and boxes were placed back in their original numbered containers until testing.

### Mechanical testing

**Fig. 1:**
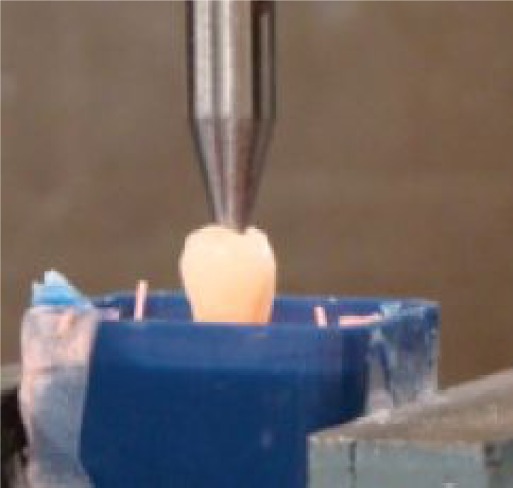
Compressive strength testing of specimens carried out using a Hounsfield testing machine. The hardened steel spike with a diameter of 3 mm was positioned at the very centre of the occlusal surface of the tooth crown between buccal and palatal cusps.

**Fig. 2:**
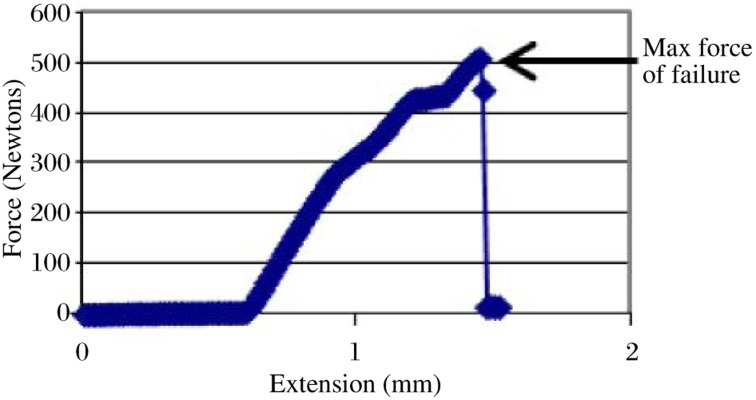
Representative dynamic compressive strength tested on a tooth sample for determination the maximum force of failure.

Compressive strength testing of specimens was performed by a Hounsfield H50KM Universal testing machine (Hounsfield Test Equipment Ltd., England) with a maximum load cell of 2,000 Newtons and the crosshead speed set at 1 mm/min. The force was applied through a hardened steel 10 mm spike with a machine rounded end with a diameter of 3 mm. This was positioned at the very centre of the occlusal surface of the tooth crown between the buccal and palatal cusps (***Fig. 1***). The applied force was continued until the tooth fractured. A force *vs* extension curve was dynamically plotted for each tooth (***Fig. 2***) and from this, the maximum force of failure in Newtons was recorded and used to determine the compressive strength in MegaPascals by the contact area of the spike applied to the tooth crown. This parameter was used for group comparisons.

### Fracture pattern

The pattern of fracture of every specimen was recorded.

### Statistical analysis

The data generated from the mechanical testing were analyzed using one-way ANOVA and Tukey tests. The level of significance to reject the null hypothesis was set at *P* < 0.05.

## RESULTS

**Fig. 3:**
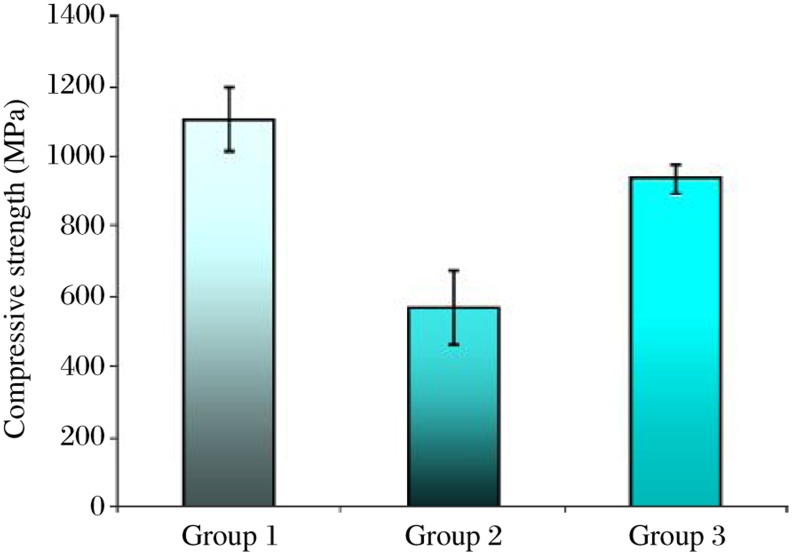
Compressive strength (MPa) of premolars in different stages of root canal therapy. Group 1: intact teeth. Group 2: access cavity and root canal preparation. Group 3: access cavity, root canal preparation, root-filled and restored with glass ionomer and composite.

Intact controls (group 1) demonstrated the greatest resistance to fracture with a mean compressive strength value of 1105.83±90.93 MPa followed by teeth that had access cavities restored (group 3) with a mean value of 936.67±44.67 MPa. Teeth with access cavities and root canal preparation but unrestored (group 2) demonstrated the least resistance to fracture with a mean value of 568.33±105.49 MPa (***Fig. 3***).

Although the compressive strength values recorded for groups 1 and 3 were statistically significantly higher than those of group 2 (*P* < 0.05), there was no significant difference between group 1 and 3.

**Fig. 4:**
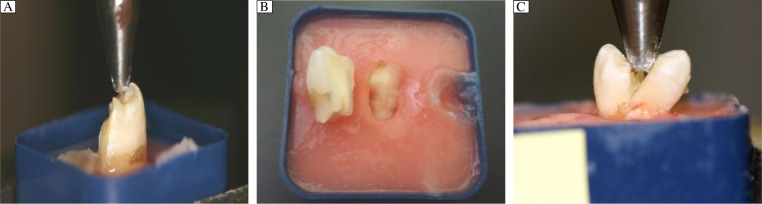
Pattern of tooth fracture. A: oblique fracture. B: crown fracture at the cementoenamel junction level. C: vertical fracture.

Three predominant fracture patterns were observed: (A) oblique fracture, (B) crown fracture at cementoenamel junction (CEJ), and (C) vertical fracture (***Fig. 4***). All oblique fractures (A) were deemed to be restorable and extended palatally. The one tooth that exhibited a crown fracture at the CEJ level (B) and all teeth with vertical fractures (C) were deemed to be non-restorable. The incidence and percentage of restorability for each group are presented in ***Table 1***.

**Table 1:** Patterns of tooth fracture in different groups.

	A	B	C	Restorable
Group 1	4	1	1	4/6 (67%)
Group 2	2	0	4	2/6 (33%)
Group 3	1	0	5	1/6 (17%)
Total	7	1	10	7/18 (39%)

Group 1: intact, Group 2: access cavity and root canal therapy preparation, Group 3: access cavity, RCT preparation, obturation, restoration with glass ionomer and composite. A: oblique fracture, B: crown fracture at the cementoenamel junction. C: vertical fracture.

## DISCUSSION

A number of studies, with or without root canal preparation, have demonstrated that if the cavity involves the marginal ridge, then the fracture resistance of the tooth is significantly reduced. Hood[11] proposed the cantilever beam model to illustrate the effect of increased depth of cavity preparation on cuspal flexure when the marginal ridges are incorporated in a cavity design. With deeper cavity preparations the length of the cusp increases and its deflection increases according to a cube of the length. However, Jantarat *et al.*[12] compared a couple of methods to measure cuspal deformation in teeth and found that cusps do not deform as simple cantilever beams. Using molar teeth with an extensive MOD cavity preparation with or without endodontic access they found that an access cavity may cause a change from predominantly flexural deformation to bulk displacement of the cusps due to a wedging apart from the cusps without deformation. It is unknown what pattern of displacement or deformation would occur if the marginal ridges remain intact.

Upper premolars were chosen for this study because the cuspal inclines render them more susceptible to force that may promote cusp fracture. Teeth with extreme curvatures, or those with 3 roots were excluded from the study. In order to limit the amount of standard deviation found when testing teeth, the teeth were placed into lots with those of similar shape. From these lots the teeth were then randomly assigned into the test groups.

The small sample size is a result of the difficulty in obtaining intact caries-free premolars with closed apices. In order to overcome the limitations of this, one-way ANOVA and Tukey tests were utilized.

This study has shown that access cavity and root canal preparations significantly reduce the fracture resistance of the premolar tooth, which is contrary to the findings of Steele & Johnson[9] and Reeh *et al.*[10] Teeth that had been obturated and then restored with glass ionomer and composite showed a reduction in fracture resistance compared to intact controls, but were not significantly different. It would seem within the confines of this experiment that simply placing an occlusal restoration after completion of RCT would be acceptable from a fracture resistance perspective. However, during the different stages of RCT the access cavity is temporarily restored. Even with just an occlusal cavity prepared and with intact marginal ridges, the tooth is significantly weaker than the intact control. A number of materials have been advocated for this and include: cotton pellet, cavit, intermediate restorative material (IRM) and glass ionomer. If the material is nothing more than a space filler, then the tooth is potentially at risk until being definitively restored. More research is indicated in this area.

Cusp fracture patterns depend on the direction and amount of force applied, and the ability of the tooth to recover from the deformation. Force may be relatively light and repetitive as in normal mastication, relatively heavy and repetitive as seen in bruxism and extremely heavy and sudden biting on a hard object or trauma.

Oblique fractures were the predominant pattern for intact teeth, with the majority deemed to be restorable. When an access cavity had been prepared, with and without restoration, vertical fractures were more commonly observed. The vertical fractures in every case would have rendered the tooth non-restorable.

In conclusion, whilst the restoration of an occlusal access cavity with glass ionomer and composite can render the tooth almost as resistant to fracture as an intact tooth, the fracture pattern was vertical and catastrophic compared to the oblique pattern seen in intact teeth.

Within the limitations of this study, upper premolar teeth that have only an occlusal access cavity are likely to be as strong as an intact tooth and do not require further cuspal protection.
